# AMPK leads to phosphorylation of the transcription factor Nrf2, tuning transactivation of selected target genes

**DOI:** 10.1016/j.redox.2019.101393

**Published:** 2019-11-27

**Authors:** Manuel Matzinger, Katrin Fischhuber, Daniel Pölöske, Karl Mechtler, Elke H. Heiss

**Affiliations:** a Department of Pharmacognosy, University of Vienna, Vienna, Austria; b Institute of Molecular Pathology (IMP), Vienna BioCenter (VBC), Vienna, Austria; c Institute of Molecular Biotechnology, Austrian Academy of Sciences, Vienna BioCenter (VBC), Vienna, Austria

**Keywords:** NFE2L2, Nrf2, AMPK, Phosphorylation, Proteomics, Transcription factor

## Abstract

The transcription factor Nrf2 (nuclear factor (erythroid-derived 2)-like 2) and the kinase AMPK (AMP-activated protein kinase) participate in the cellular adaptive response to redox or energy stress. Despite accumulating evidence for positive cooperativity between both proteins, information about direct post-translational modification of Nrf2 by AMPK in living cells is scarce. Here, MS-based analysis of immunoprecipitated Nrf2 revealed serine 374, 408 and 433 in human Nrf2 to be hyperphosphorylated as a function of activated AMPK. A direct phosphate-transfer by AMPK to those sites was indicated by *in vitro* kinase assays with recombinant proteins as well as interaction of AMPK and Nrf2 in cells, evident by co-immunoprecipitation. Mutation of serine 374, 408 and 433 to alanine did not markedly affect half-life, nuclear accumulation or induction of reporter gene expression upon Nrf2 activation with sulforaphane. However, some selected endogenous Nrf2 target genes responded with decreased induction when the identified phosphosites were mutated, whereas others remained unaffected. Notably, the genes susceptible to the mutation of the phosphorylation sites in Nrf2 consistently showed reduced induction in AMPKα1 −/−cells. Overall, our data reveal AMPK-triggered phosphorylation of Nrf2 at three serine residues, apparently determining the extent of transactivation of selected target genes.

## Highlights

• Serine 374, 408 and 433 of Nrf2 are AMPK-dependently phosphorylated in cells.
• *In vitro* kinase-and CoIP assays strongly point to AMPK as a direct Nrf2 kinase.
• These phosphosites are not crucial for stability or nuclear accumulation of Nrf2.
• They, however, seem to modulate transactivation of a subset of Nrf2 target genes.

## Graphical abstract

**Figure:**
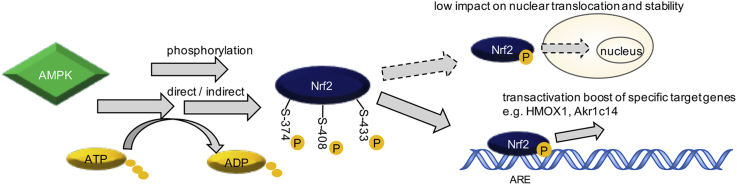

## 1 Introduction

Nuclear factor (erythroid-derived 2)-like 2 (Nrf2) is a stress responsive basic leucine zipper transcription factor, known as master regulator of cellular (redox) homeostasis and detoxification. It is ubiquitously expressed and regulates more than 200 genes with so-called antioxidant response elements (ARE) in the regulatory region, including genes of the antioxidant defense, detoxification, proteostasis or for metabolic enzymes [1]. Under basal conditions the steady state levels of Nrf2 are low, due to interaction of Kelch-like ECH-associated protein 1 (Keap1) with the Neh2 degron or of β-transducin repeat containing protein 1 (β-TrCP) with the Neh6 degron of Nrf2, which is accompanied by Nrf2 ubiquitylation and proteasomal degradation [2,3]. Upon diverse stressful insults, ubiquitination ceases, Nrf2 accumulates and translocates to the nucleus. There it heterodimerizes with small musculoaponeurotic fibrosarcoma (Maf) proteins and binds to ARE sequences in regulatory DNA elements, which leads to induced transcription of target genes [4]. Moreover, post-translational modifications (PTMs) of Nrf2, including phosphorylation or acetylation, influence nuclear translocation or transactivation properties [5].

5′-AMP-activated protein kinase (AMPK), a heterotrimeric serine/threonine kinase composed of a catalytic α as well as regulatory β and γ subunits, constitutes a major switch to maintain energy homeostasis. AMPK activity rises in the presence of high AMP/ATP or ADP/ATP ratios, elevated calcium levels, or reduced glucose supply. Activation is primarily regulated allosterically (by binding of AMP at the γ or small molecules at the β subunit) or covalently via phosphorylation at Thr 172 by upstream kinases and by inhibition of dephosphorylation at Thr 172 (reviewed in Ref. [6]).

Activated AMPK increases catabolic and decreases anabolic activities, leading to increased ATP generation and reduced ATP consumption [[7], [8], [9]]. Besides its role in energy homeostasis, activated AMPK has also been linked to reduced inflammation, decreased redox stress or restricted proliferation [[10], [11], [12]].

Activated Nrf2 or AMPK signaling elicit strikingly overlapping phenotypic cellular or organismal responses and are concomitantly activated by several small (natural) molecules, indicating cooperativity [[13], [14], [15]]. Accordingly, an AMPK-driven boost of the Nrf2 signaling axis has already been confirmed in several studies (e.g. Refs. [16,17]). While most of those investigations showed an indirect influence of AMPK, mainly via signal relay over the p62/autophagy/Keap1- (e.g. Ref. [18]) or the glycogen synthase kinase 3 β (GSK3 β)/β-TrCP- axes (e.g. Ref. [19]), only one study so far demonstrated direct phosphorylation of Nrf2 by AMPK in an *in vitro* enzyme assay at position Ser 558 (human Nrf2) [20]. Therefore, we set out to investigate (additional) AMPK-dependent phosphorylation sites at Nrf2 occurring in living cells and to shed light onto their functional relevance for Nrf2 signaling.

## 2 Material and methods

### 2.1 Cells, chemicals, antibodies and plasmids

Mouse embryonic fibroblasts (MEF wt/Nrf2 −/−/Keap1−/− and MEF wt/AMPKα1 −/−) were kind gifts from Thomas Kensler (University of Pittsburgh, USA) and Benoit Viollet (Institut Cochin, France) respectively. Human embryonic kidney cells (HEK293) cells were obtained from ATCC (Manassas, VA, USA). Media, serum and supplements for cell culture were purchased from Lonza and Invitrogen.

Sulforaphane (Sfn) and cycloheximide (CHX) were purchased from Sigma –Aldrich (Vienna, Austria), A769662 and MG132 came from ApexBio (Houston, TX, USA), dorsomorphin (= Compound C) was obtained from Abcam (Cambridge, UK). Hoechst 33342, Lipofectamine™ LTX and PLUS™ Reagent were from Thermo Fisher (Rockford, IL, USA). DNase I (RNase free, 20 u/μL within the peqGOLD Total RNA kit) was from VWR (Vienna, Austria). Recombinant GST-tagged NFE2L2 Protein (GST Nrf2, #H00004780-P01) was obtained from Abnova (Taipei City, Taiwan) and recombinant AMPK α1/β1/μ1 (#P47-10H 10) from Signal Chem (Richmond, Canada). Trypsin gold and chymotrypsin for proteolytic digests were purchased from Promega (Mannheim, Germany) and lysyl endopeptidase (LysC) was from Wako (Neuss, Germany).

The antibodies raised against AMPKα (#2532), acetyl-CoA carboxylase *P*-Ser79 (PACC, #3661), myc tag (#2276) and lamin (#12586) as well as the horseradish peroxidase coupled secondary antibodies (#7074S, #7076S) were purchased from Cell Signaling (Frankfurt am Main, Germany) and used in 1:1000 dilutions. The anti - hemeoxygenase 1 (HMOX1) came from Enzo Life Sciences (#ADI-SPA-895F) and was used in a 1:500 dilution; the anti‐actin antibody (#69100) was obtained from MP Biologicals (Illkirch, France) and was used in a 1:5000 dilution. The antibodies detecting green fluorescent protein (GFP; sc-9996) and gamma glutamyl cysteine ligase (GCLC, γ-GCSc, #sc-166382) were from Santa Cruz (Heidelberg, Germany) and used in 1:200 dilution, and the MA (magnetic agarose) GFP-Trap® came from Chromotek (Planegg-Martinsried, Germany).

The mammalian expression vectors pcDNA3-EGFP-C4-Nrf2 (EGFP-WT-Nrf2) #21549, NC16 pcDNA3.1 FLAG NRF2 #36971, pCIP-AMPKα1_WT #79010, pEGFP-C1-PRKAA1 #30305 and pcDNA3-myc3-β-TrCP #20718 were obtained from Addgene (Watertown, MA, USA). The triple serine-alanine mutant form of Nrf2 (EGFP-TM-Nrf2) was gradually generated using the Q5® Site-Directed Mutagenesis Kit from New England Biolabs (Frankfurt am Main, Germany). Desired mutations were confirmed by sequencing (Microsynth, Balgach, Switzerland). The EGFP expression vector was from ClonTech (Mountain View, CA, USA), and ARE (mGST)/luciferase (ARE-LUC) reporter gene construct was a kind gift from Donna Zhang (University of Arizona, USA). Plasmid amplification and isolation followed standard procedures.

### 2.2 Cell cultivation, transfection and compound treatment

HEK293 or MEF cells were maintained at 37 °C and 5% CO_2_ in Dulbecco's modified essential medium (DMEM), supplemented with 10% (v/v) fetal calf serum, 2 mM glutamine, 100 U/mL benzylpenicillin and 100 μg/mL streptomycin.

Transfection was performed at 60–70% cell confluency in 10 cm dishes. For HEK293 cells the calcium phosphate precipitation method was used [21]. MEF cells were transfected using Lipofectamine™ LTX and PLUS™ Reagent according to the manufacturer's instructions but using serum free DMEM supplemented with 2 mM glutamine, 100 U/mL benzylpenicillin and 100 μg/mL streptomycin instead of Opti -MEM®.

Unless stated otherwise, cells were treated with A769662 (50 μM) and Compound C (10 μM) for activation and inhibition of AMPK, respectively. For pharmacological activation and stabilization of Nrf2, cells were exposed to sulforaphane (Sfn) (5 μM) or the proteasomal inhibitor MG132 (10–20 μM), respectively. *De novo* protein synthesis was inhibited with cycloheximide (30–100 μM). DMSO was used as vehicle control, kept at the same concentration in all samples of one experiment and never exceeded 0.1% (v/v).

### 2.3 Immunoprecipitation (IP)

After protein extraction with RIPA buffer (10 mM Tris/Cl pH 7.5, 150 mM NaCl, 0.5 mM EDTA, 0.1% SDS, 1% Triton X-100, 1% Deoxycholate supplemented with: Roche cOmplete™ Mini Protease Inhibitor Cocktail, 1 mM PMSF, 1 mM NaF, 1 mM Na_3_VO_4_, 5 mM sodium butyrate, 2.5 mM MgCl2 and 90 u DNase I prior to use) and sonication, equal total protein amounts (range of 4 mg) were incubated with 25 μL GFP-Trap® magnetic agarose beads and worked up by washing using RIPA buffer and finally by washing with a detergent free buffer (10 mM Tris/Cl pH 7.5; 50 mM NaCl). For elution followed by immunoblot analysis, the beads were incubated in 1 x SDS buffer at 95 °C for 5 min.

### 2.4 SDS polyacrylamide gel electrophoresis and immunoblot analysis

They were performed as previously described [22]. To separate nuclear from cytosolic proteins, cells were first washed with cold PBS and then exposed to buffer 1 (10 mM HEPES pH 7.5, 0.2 mM EDTA, 10 mM KCl, 1% NP40 (IGEPAL®), 1 mM DTT, 0.5 mM PMSF, CompleteTM (Roche, Switzerland)). Cells were scraped off and transferred into a microtube and incubated for 15 min on ice, with vigorous vortexing every 2–3 min, and centrifuged for 5 min at 11,000 g. The supernatant contained the cytosolic fraction. Pellets were washed once with buffer 1 and were then resuspended in buffer 2 (20 mM HEPES pH 7.5, 1.1 mM EDTA, 420 mM NaCl, 1 mM DTT, PMSF and CompleteTM (Roche, Switzerland)), incubated on ice for 15 min with vigorous vortexing every 2–3 min, followed by centrifugation for 5 min at 11,000 g. The supernatant contained nuclear proteins. Successful separation of cytosolic and nuclear fractions was routinely validated by immunoblotting of tubulin (cytosolic marker) and lamin (nuclear marker), respectively.

### 2.5 Isolation of RNA, reverse transcription and quantitative polymerase chain reaction (q-PCR)

They were performed as previously described [17]. QuantiTect primer kits for murine *Gclc*, aldoketoreductase (*Akr*) 1c14, NAD(P)H quinone dehydrogenase (*Nqo*)1 and hypoxanthine-phospho-ribosyl transferase (*Hprt*) were obtained from Qiagen (Hilden, Germany). The murine *Hmox1* primers (fwd: AAGCCGAGAATGCTGAGTTCA, rev: GCCGTGTAGATATGGTACAAGGA) were custom synthesized by Thermo Fisher.

### 2.6 Luciferase reporter gene assay

It was essentially performed as previously described [23].

### 2.7 Confocal imaging

Cells were grown on coated (0.2% gelatin, 30 min, 37 °C) coverslips. After transfection with expression plasmids for EGFP-tagged WT or TM-Nrf2 and treatment with sulforaphane (5 μM, 4 h) nuclei were stained with Hoechst 33342 (1 μg/mL) for 20 min. After fixation with 4% methanol-free formaldehyde solution in PBS for 10 min at 37 °C and mounting, samples were analyzed with an ACS APO 63x/1.30 oil immersion objective using a Leica DMi8 inverted confocal microscope and LAS X (3.1.2) software.

### 2.8 *In vitro* kinase assay

The employed protocol was adapted from literature [20,24]. Briefly, 0.6 μg recombinant AMPK α1/β1/μ1 and 1.6 μg GST Nrf2 were mixed in a total volume of 120 μL of a 20 mM HEPES pH 7.4 buffer containing 200 μM ATP, 1 mM DTT, 5 mM MgCl_2_, 1 mM EDTA, 200 μM NaF and a varying concentration of 0–100 μM AMP. After an incubation of 30 min at 30 °C, samples were prepared for LC - MS analysis.

### 2.9 Liquid chromatography – mass spectrometry (LC – MS) analysis

IP samples were eluted by resuspension of beads (GFP-Trap®) in 100 mM ammonium bicarbonate buffer and incubated with 400 ng of LysC for 4 h at 37 °C. The thereby pre-digested IP-samples or recombinant protein mixtures (*in vitro* kinase assay) were reduced using 6.5 mM TCEP (tris(2-carboxyethyl)phosphine) (30 min; 56 °C; shaking). Alkylation with MMTS (methyl methanethiosulfonate) for IP samples or IAA (2-Iodoacetamide) for recombinant proteins (30 min; RT, w/o light) was followed by an enzymatic digest either by trypsin (o/N; 37 °C) or by chymotrypsin (5 h; 25 °C). For IP samples a fixed amount of 200 ng protease was added, for recombinant proteins an enzyme to protein ratio of 1:50 was chosen. The digestion was stopped by addition of TFA (trifluoracetic acid) to a final concentration of 1% (v/v).

For NanoLC - MS analysis digested peptides were separated with a Dionex UltiMate 3000 HPLC RSLC nano-system coupled to an QExactive HF Orbitrap mass spectrometer via Proxeon nanospray source (both: Thermo Fisher Scientific). Peptides were loaded onto a trap column (Thermo Fisher Scientific, PepMap C18, 5 mm × 300 μm ID, 5 μm particles, 100 Å pore size) at a flow rate of 25 μL min^−1^ using 0.1% TFA as mobile phase. After 10 min, the trap column was switched in line with the analytical column (Thermo Fisher Scientific, PepMap C18, 500 mm × 75 μm ID, 2 μm, 100 Å). Peptides were eluted using a flow rate of 230 nl min^−1^, with the following gradient over 200 min (shortened to 80 min for recombinant proteins): 0–10 min 2% buffer B, followed by an increasing concentration of buffer B up to 35% until min 60 (recombinant proteins) to min 180 (IP samples). This is followed by a 5 min gradient from 35–95% B, washing for 5 min with 95% B, followed by re-equilibration of the column at 30 °C (buffer B: 80% ACN, 19.92% H_2_O and 0.08% TFA, buffer A: 99.9% H_2_O, 0.1% TFA).

The mass spectrometer was operated in a data-dependent mode, using a full scan (*m*/*z* range 380–1500, nominal resolution of 60,000, target value 1E6) followed by MS/MS scans of the 10 most abundant ions. MS/MS spectra were acquired using an NCE (normalized collision energy) of 27, an isolation width of 2.0 *m*/*z*, a resolution of 30.000 and the target value was set to 1E5. Precursor ions selected for fragmentation (exclude charge state 1, 7, 8, >8) were put on a dynamic exclusion list for 20 s. Additionally, the minimum AGC target was set to 2E4 and intensity threshold was calculated to be 8E4. The peptide match feature was set to preferred and the exclude isotopes feature was enabled.

MS data were analyzed with the help of Thermo Proteome Discoverer (2.1.081). Peptide identification was performed by MS Amanda (2.0.0.9849) [24]. The peptide mass tolerance was set to ±5 ppm and the fragment mass tolerance to ±0.03 Da. The maximal number of missed cleavages was set to 2. The result was filtered to 1% FDR (false discovery rate) on peptide level using Percolator algorithm integrated in Thermo Proteome Discoverer. The localization of the post-translational modification sites within the peptides was performed using the ptmRS (1.4.6548.19923) [25] node. Quantification was performed label free using apQuant (2.0.65.20439) [26]. For IP samples, the search was performed against a full human database (swissprot, including contaminants, containing in total 20,345 proteins), for the *in vitro* kinase assay a database containing both recombinant proteins and contaminants (total 366 proteins) was used.

### 2.10 Statistical analysis

Unless stated otherwise, at least three independent biological replicates were performed for all experiments. The bar graphs depict mean +SD (standard deviation). Two groups were compared via Student's *t*-test or the Welch's *t*-test by using GraphPad Prism 6 software. Differences considered as significant (P < 0.05) are labelled with a * within graphs.

## 3 Results

### 3.1 Activated AMPK leads to phosphorylation of Nrf2

**Fig. 1:**
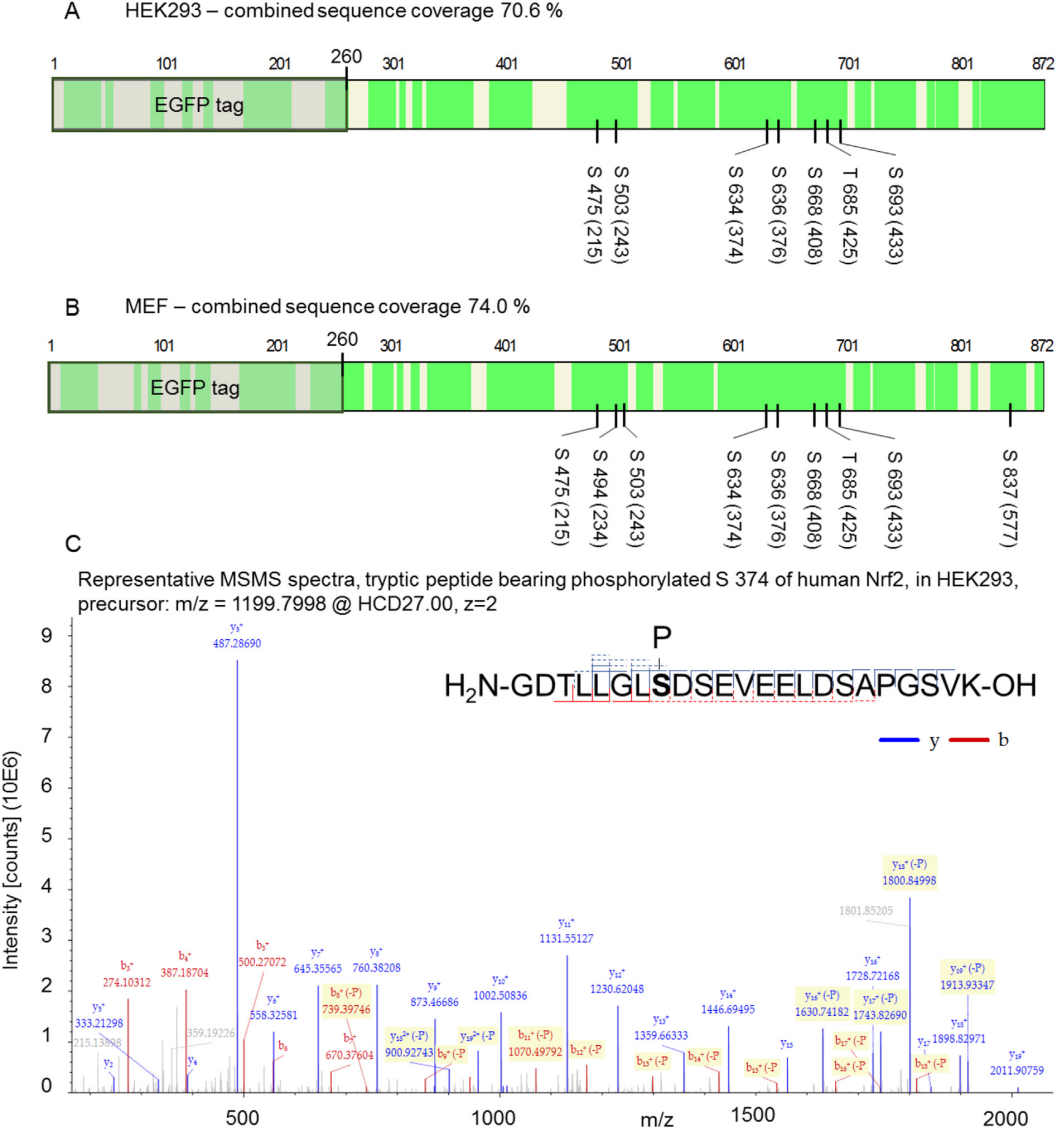
**Identified phosphorylation sites in Nrf2 after immunoprecipitation from cell lysates and subsequent MS analysis**. HEK293 **(A)** or MEF (wt) **(B)** were transiently transfected with an expression plasmid for human EGFP-WT-Nrf2 and treated with proteasome inhibitor (and AMPK activator) for 4 h. After cell lysis, Nrf2 was pulled down by a GFP-Trap and prepared for MS analysis as described in the method section. Covered sequence areas are marked in green and reproducibly found phosphorylation sites at S or T residues within the EGFP-WT-Nrf2 fusion protein are indicated (in brackets: corresponding position in native human Nrf2). **(C)** Representative example of a MS/MS spectra from a phospho-peptide, showing the resulting fragments (b and y series) after HCD fragmentation (27 NCE), at position 8 serine is phosphorylated. The b series is shown in red, the y series is shown in blue (dashed lines: fragments with modification loss, more than one line: found in different charge states as indicated in the spectra). (For interpretation of the references to colour in this figure legend, the reader is referred to the Web version of this article.)

**Fig. 2:**
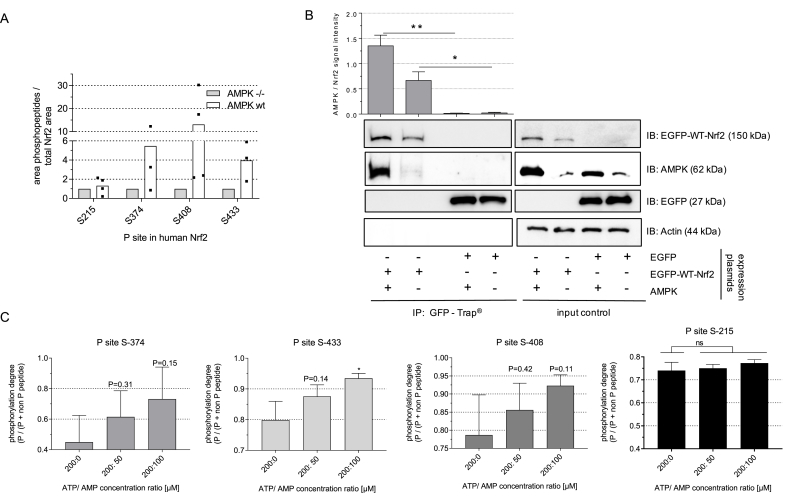
**Nrf2 is AMPK-dependently phosphorylated at serine 374, 408 and 433, interacts with AMPK and is an AMPK substrate**. (**A)** AMPK wt or AMPKα1 −/− MEF were transiently transfected with EGFP-WT-Nrf2 and treated with AMPK activator A769662 (50 μM, 4 h) and proteasome inhibitor (MG132, 20 μM). Accumulated Nrf2 was pulled down from cell lysates and after tryptic/chymotryptic digestion subjected to LC-MS analysis and screened for AMPK-dependent phosphosites. For S 374, 408 and 433 the relative amount (area by LFQ) of phosphosite specific peptides normalized to the total amount of Nrf2 is shown for WT and AMPKα1 −/−MEF (S 215 depicted as presumably AMPK independent site). Dots show the obtained area in single biological replicates, bars indicate the mean. **(B)** HEK cells were transfected with expression plasmids for EGFP-WT-Nrf2, PRKAA1 (AMPK) or EGFP as indicated. After stabilization of Nrf2 (via proteasome inhibition), lysis and pulldown via GFP-Trap®, eluates as well as an aliquot (20 μg protein) of the unprecipitated lysate (input control) were immunoblotted for GFP (= EGFP-WT-Nrf2 or EGFP) or AMPKα1. Representative blots and compiled densitometric analyses (AMPK signal/GFP signal) are depicted (n = 3, unpaired Student's t-test, two tailed, α = 0.05, *P < 0.05). **(C)** Recombinant AMPK and Nrf2 were subjected to an *in vitro* kinase assay with varying ratios of ATP to AMP (as indicated, fixed ATP concentration of 200 μM). After termination of the reaction, Nrf2 was prepared for and subjected to MS analysis for phosphosite identification and quantification. The obtained area of the most abundant peptide of Nrf2 for each indicated phosphosite was normalized as follows: [area P peptide/(area P peptide + area same peptide without phosphorylation)], which gives the degree of phosphorylation. A value of 1 would indicate that 100% of the detected peptide was phosphorylated at that position and no unphosphorylated peptide was found. (n = 3, mean +SD, unpaired Student's t-test, two tailed, α = 0.05, *P < 0.05, ns: not significant).

Aiming to track possible AMPK-dependent phosphorylation sites of Nrf2 in living cells, we pulled down stabilized EGFP-WT-Nrf2 from lysates of transfected cells and analyzed its phosphorylation pattern by MS analysis after (chymo)tryptic digest. In order to investigate AMPK-dependency, we compared results obtained from cells with and without activated AMPK, i.e (i) HEK cells treated with AMPK activator (A769662) or inhibitor (Compound C), as well as (ii) wt or AMPKα1 −/− MEF cells treated with AMPK activator. Data analysis from the HEK293 setting uncovered a total number of 12 serine or threonine phosphosites in Nrf2, of which 7 were found in all three performed biological replicates (Fig. 1A). In transfected MEF cells, 12 serine or threonine phosphosites were detected for Nrf2, of which 9 serine residues were phosphorylated in all replicates (Fig. 1B). Fragmentation patterns of each phosphorylated peptide enabled precise localization of the respective phosphosites (an example is shown in Fig. 1C). Label free quantification of the obtained MS datasets finally revealed three serine residues, i.e. S 374, S 408 and S 433 in human Nrf2, with a reproducibly enhanced phosphorylation degree in cells with active AMPK compared to cells with reduced or no AMPK activity (Fig. 2A, Supplemental Figs. 1A and 1B). The presumably AMPK-independent phosphosite at S 215 was evenly phosphorylated, irrespective of enhanced or lacking AMPK activity.

To examine Nrf2 as a potential direct AMPK substrate, we checked interaction between AMPK and Nrf2 by co-immunoprecipitation from cell lysates and performed an *in vitro* kinase assay with pure recombinant proteins. As seen in Fig. 2B, endogenous AMPK co-immunoprecipitates with pulled down EGFP-Nrf2, an effect which is pronounced in the case of overexpressed AMPK. In control lysates carrying EGFP instead of EGFP-Nrf2 hardly any AMPK signal after pulldown of GFP could be observed, indicating Nrf2-specific coelution of AMPK. The interaction between Nrf2 and AMPK was further corroborated by reverse co-immunoprecipitation of Nrf2 after specific pulldown of AMPK (Supplemental Fig. 1C). AMPK also popped up as potential interaction partner of Nrf2 within unbiased proteomic analyses of endogenous proteins pulled down together with Nrf2, however, with low abundancy compared to Keap1 or Maf proteins, suggesting weak, transient or sporadic interaction (unpublished data). In an *in vitro* kinase assay (Fig. 2C) all identified three phosphorylation sites reproducibly showed an increased phosphorylation degree with growing AMPK activity (increased AMP/ATP ratio), reaching significance for S 433. Again, phosphorylation at S 215 was not altered upon increased AMPK activity in the *in vitro* assay.

Overall, S 374, S 408 and S 433 of Nrf2 are phosphorylated in an AMPK-dependent, if not AMPK - catalyzed manner in living cells.

### 3.2 The identified AMPK-dependent phosphosites do not markedly contribute to Nrf2 stability

**Fig. 3:**
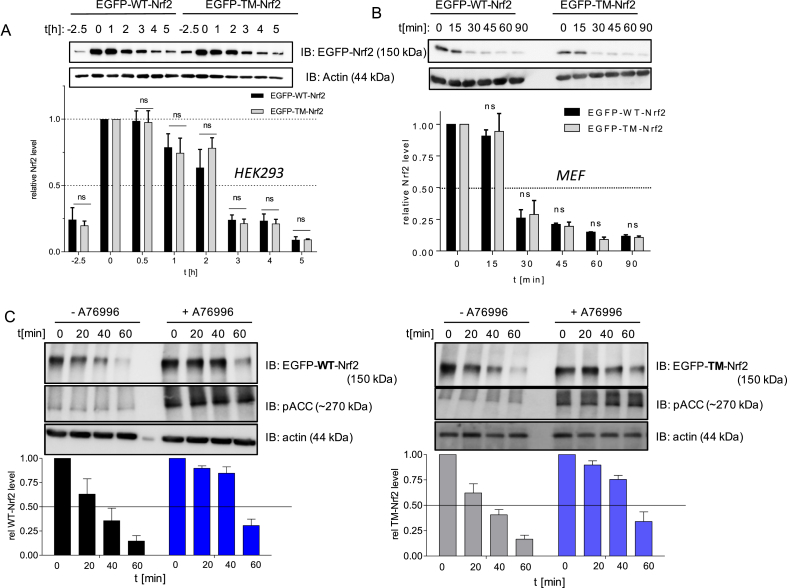
**The identified AMPK-dependent phosphosites in Nrf2 do not markedly influence half-life of Nrf2 in wt cells. (A)** HEK293 were transfected with EGFP-tagged WT or TM-Nrf2 expression plasmids and treated with MG132 (20 μM) for 2.5 h (t = −2.5 to 0) to accumulate Nrf2. After washout of MG132 the translation inhibitor cycloheximide (100 μM) was added (time point 0) for another incubation period of 1–5 h as indicated. Cell lysates were subjected to immunoblot analysis for EGFP-Nrf2 (via α-GFP antibody) or actin. Representative blot and compiled densitometric evaluations (below) for EGFP-Nrf2/actin (related to signal at t = 0) are depicted. (n ≥ 3, mean +SD, unpaired Student's t-test, two tailed, α = 0.05, *P < 0.05, ns: not significant). **(B)** Wt MEF were transfected with EGFP-tagged WT or TM-Nrf2 expression plasmids and treated with MG132 (10 μM) for 1 h to accumulate Nrf2. After washout, cycloheximide (30 μM) was added (time point 0) for the indicated periods of time. Cell lysates were subjected to immunoblot analysis for EGFP-Nrf2 (via α-GFP antibody) or actin. Representative blots and compiled densitometric evaluations (below) for EGFP-Nrf2/actin (related to signal at t = 0) are depicted. (n = 3, mean +SD, unpaired Student's t-test, two tailed, α = 0.05, *P < 0.05, ns: not significant).**(C)** Wt MEF were transfected with EGFP-tagged WT- or TM-Nrf2 expression plasmids and treated with MG132 (10 μM) and DMSO or A769662 (50 μM) for 1 h. After washout of MG132 the translation inhibitor cycloheximid (30 μM) was added (time point 0) in the presence of A769662 (50 μM) (+) or DMSO (−) for the indicated period of time. Cell lysates were subjected to immunoblot analysis for EGFP, pACC or actin. Representative blots and compiled densitometric evaluations for DMSO and A769662-treated cells (EGFP-WT- or EGFP-TM-Nrf2/actin, related to signal at t = 0) are depicted (n = 3, mean +SD).

**Fig. 4:**
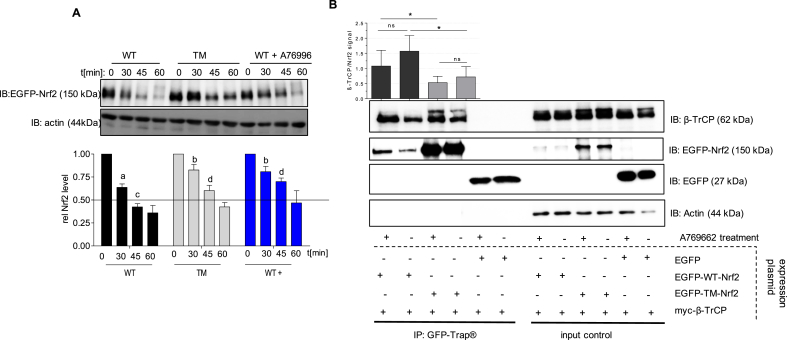
**Mutation of the AMPK-dependent phosphosites to alanine stabilizes Nrf2 in Keap1−/− cells and impedes interaction with β-TrCP. (A)** Keap1−/− MEF were transfected with EGFP-tagged WT- or TM-Nrf2 expression plasmids, pretreated with A76996 (50 μM, 1h) as indicated and then exposed to cycloheximid (30 μM) for different periods of time. Cell lysates were subjected to immunoblot analysis for EGFP or actin. Representative blots and compiled densitometric evaluations (EGFP-WT/TM-Nrf2/actin, related to signal at t = 0) are depicted (n = 3, mean +SD, unpaired Student's t-test, two tailed, α = 0.05, different superscript letters indicate differences with p < 0.05). **(B)** HEK cells were transfected with an expression plasmid for myc-tagged β-TrCP together with constructs encoding EGFP-WT-Nrf2, the triple mutated version EGFP-TM-Nrf2, or EGFP as indicated. Cells were treated with vehicle (DMSO) or A769662 (50 μM) 1 h prior to addition of MG132 (20 μM) for further 3 h to stabilize Nrf2, which was pulled down using GFP-Trap®. (Co) precipitated proteins as well as input controls were immunoblotted for myc (=β-TrCP) or GFP (=EGFP-WT-/EGFP-TM-Nrf2 or EGFP) as indicated. Representative blot pictures and densitometric evaluations (relative myc (β-TrCP)/GFP (Nrf2) signal) are depicted. (n = 6, mean +SD; *P < 0.05, Welch's *t*-test, two tailed, α = 0.05).

To address the functional relevance of the identified phosphosites we created a triple mutated version of Nrf2 (EGFP-TM-Nrf2), in which S 374, S 408 and S 433 were replaced by alanine. Since the affected serine residues are close to the Neh6 degron, including the β-TrCP binding sites 343-DSGIS-347 and 382-DSAPGS-387 [25] of Nrf2 which are involved in proteasomal degradation, we were prompted to first check the influence of the phosphosites on Nrf2 stability. For this, EGFP-WT- and EGFP-TM-Nrf2 were expressed and accumulated by proteasome inhibition via MG132 treatment in HEK or MEF cells. After washout, protein *de novo* synthesis was stopped by addition of cycloheximide which allowed monitoring of the time-dependent decay of existent Nrf2. Both EGFP-WT- and EGFP-TM-Nrf2 displayed similar kinetics of degradation in the HEK as well as in wt MEF cell system (Fig. 3A and B). This finding is in line with an unaltered ubiquitination between WT and mutant Nrf2, seen in MG132-treated transfected MEF (Supplemental Fig. 2). Concomitant pharmacological AMPK activation by the AMPK activator A769662 (evident by successful phosphorylation of S79 in acetyl-CoA carboxylase) achieved stabilization of both EGFP-WT- and EGFP-TM-Nrf2 to a comparable extent (Fig. 3C). The Nrf2 stabilization upon AMPK activation by A769662 was completely lost in isogenic AMPKα1 −/− MEF, excluding off-target effects (Supplemental Fig. 3). In order to more specifically examine the role of AMPK activation or the identified phosphosites for the β-TrCP - regulated Nrf2 degradation, i.e. unmasked from the presumably dominating canonical Keap1-triggered depletion, we transfected Keap1 −/− MEF with EGFP-WT- or TM-Nrf2. Monitoring the Nrf2 decay in the presence and absence of AMPK activation again revealed stabilization of WT-Nrf2 by AMPK activation and an already extended half-life for the mutated form (Fig. 4A). TM- Nrf2 was not susceptible to stabilization by AMPK activation (Supplemental Fig. 4). Combined densitometric evaluation of three independent co-immunoprecipitation experiments (β-TrCP/Nrf2 signal) further indicated that the interaction of Nrf2 with β-TrCP is tendentially alleviated after pharmacological AMPK activation but also markedly reduced upon mutation of the three identified phosphorylation sites (Fig. 4B).

In conclusion, activation of AMPK can prolong abundance of Nrf2. The identified phosphosites at S 374, S 408 and S 433 in Nrf2 do hereby not play a predominant role in wt cells. Only in a Keap1-negative background the mutated form of Nrf2 shows a prolonged half-life compared to WT-Nrf2, in line with reduced binding to β-TrCP, and no further stabilization upon AMPK activation. This can be due to conformational constraints after the triple S to A replacement impairing Nrf2/β-TrCP interaction and/or lacking phosphorylation that may be required to mediate AMPK-dependent protection from degradation via GSK/β-TrCP.

### 3.3 The identified AMPK-dependent phosphosites do not influence the ability of Nrf2 to enter the nucleus or to transactivate exogenous reporter gene expression, but boost expression of selected endogenous Nrf2 targets

**Fig. 5:**
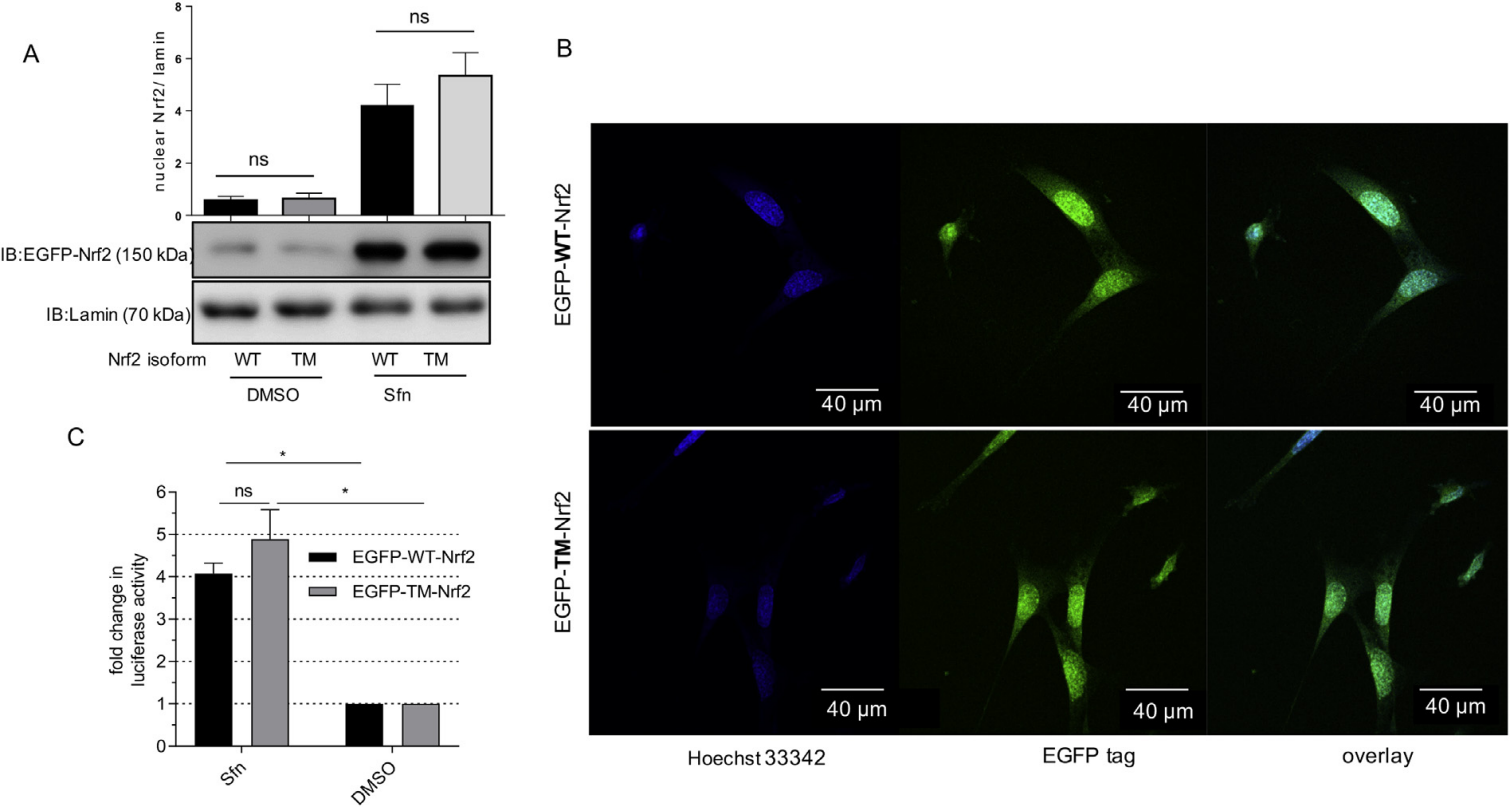
**The identified AMPK-dependent phosphosites in Nrf2 do not affect nuclear translocation or transactivation of reporter gene expression.** MEF were transfected with EGFP-tagged WT- or TM-Nrf2 and treated with DMSO as solvent control or Sfn (2 h, 5 μM). **(A)** Then nuclear fractions were prepared and blotted for GFP (=EGFP-WT- or EGFP-TM-Nrf2) and lamin. Representative blots and densitometric evaluation of three independent experiments are depicted. **(B)** Alternatively, cells were fixed. After staining nuclei with Hoechst 33342 cells were viewed under a confocal microscope (ex/em = 405/465 nm for Hoechst dye (nuclei) and ex/em = 488/520 nm (EGFP-Nrf2)). Only Sfn-treated transfected cells are depicted. **(C)** MEF Nrf2 −/− cells were transfected with EGFP-tagged WT or TM-Nrf2 expression plasmid together with an ARE-dependent luciferase reporter construct (ARE-LUC). Transfected cells were treated with DMSO or Sfn (5 μM) for 40 h before luciferase activity was determined. The bar graph depicts compiled data of at least three independent experiments expressed as fold luciferase induction by Sfn compared to DMSO-treated control cells (**A**, **C**: n ≥ 3, mean +SD, unpaired Welch's *t*-test, two tailed, α = 0.05, *P ≤ 0.05, ns: not significant).

**Fig. 6:**
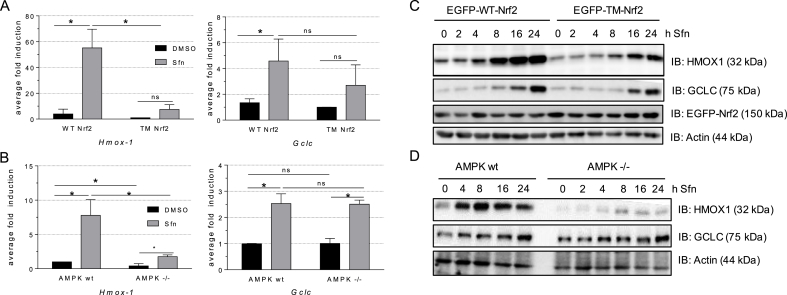
**AMPK-dependent phosphosites in Nrf2 enhance expression of selected endogenous Nrf2 target genes. (A)** MEF Nrf2 −/− cells transfected with EGFP-WT- or EGFP-TM-Nrf2 and **(B)** wt or AMPKα1 −/− MEF were treated with DMSO as vehicle control or sulforaphane (Sfn, 5 μM) for 4 h. RNA was extracted and analyzed for abundance of *Hmox1* and *Gclc* mRNA by qPCR (*Hprt* as reference gene). Bars depict compiled relative expression levels. (n = 3; mean +SD, unpaired Student's or Welch's *t*-test, α = 0.05, *P ≤ 0.05, ns: not significant. **(C)** MEF Nrf2 −/− cells transfected with EGFP-WT- or EGFP-TM-Nrf2 and **(D)** wt or AMPKα1 −/− MEF were treated with Sfn (5 μM) for the indicated periods of time. Proteins were extracted and subjected to immunoblot analysis for HMOX1, GCLC, GFP (=EGFP-WT- or EGFP-TM-Nrf2) and actin as loading control. The depicted blots are representative for three independent biological replicates with consistent results.

Next we addressed signaling behavior of WT and mutant Nrf2 in transfected cells upon treatment with sulforaphane (Sfn), a small natural molecule known to activate both Nrf2-and AMPK signaling [26,27]. Nuclear accumulation of Sfn-activated EGFP-tagged WT- and TM-Nrf2 was comparable, as evident by assessing cytosolic and nuclear WT and TM-Nrf2 by immunoblot or by using confocal microscopy (Fig. 5A and B). Expression of EGFP-WT- or EGFP-TM-Nrf2 in Nrf2 −/− MEF allowed assessment of their transactivating capacity without interference of endogenous Nrf2. Both Nrf2 forms hereby achieved equivalent induction of a transfected ARE-dependent luciferase reporter gene upon activation with Sfn (Fig. 5C), stressing general functionality of the triple mutant. To further assess transactivation of endogenous Nrf2 target genes, we next investigated the expression of *Hmox1* (heme oxygenase 1) and *Gclc* (Glutamate-cysteine ligase catalytic subunit). Whereas *Hmox1* induction was significantly reduced in cells carrying the triple mutant Nrf2 compared to cells carrying EGFP-WT-Nrf2, *Gclc* was induced to the same extent by both Nrf2 versions after Sfn exposure (Fig. 6A). Notably, this picture of a gene-selective alleviation of target gene induction by EGFP-TM-Nrf2 was mirrored when comparing wt with AMPKα1 −/− cells carrying endogenous Nrf2, speaking against a potential conformational phosphorylation/AMPK - independent issue with the triple mutant. Whereas Sfn-mediated *Gclc* induction was not affected by lack of AMPK, *Hmox1* was significantly less expressed in AMPKα1 −/− cells (Fig. 6B). Consistent with a distinct susceptibility of Nrf2 targets to AMPK, an AMPK inhibitor significantly blunted induction of *Hmox1* but not of *Gclc* in Sfn-treated MEF in an AMPK-dependent manner (Supplemental Fig. 5A). Nuclear levels of Nrf2 were hereby comparable in wt and AMPK −/− cells after Sfn exposure, excluding altered Nrf2 abundance as possible explanation for the observed difference in this experimental setting (Supplemental Fig. 5B). The pattern seen for relative mRNA levels was also reflected on protein level. Whereas HMOX1 accumulation after Sfn treatment is diminished, GCLC shows similar induction in EGFP-TM-Nrf2 transfected Nrf2 −/− or in AMPKα −/− cells, when compared to EGFP-WT-Nrf2 transfected Nrf2 −/− cells and AMPK wt cells, respectively (Fig. 6C and D). Two additionally selected Nrf2 target genes, *Nqo1* and *Akr1c14*, underlined an apparently gene/context-specific regulation of the Nrf2 transcriptome by AMPK and phosphorylation of Nrf2 at S 374, 408 and 433, respectively (Supplemental Figs. 6A and B). Induction of *Nqo1* mRNA was unaffected, whereas *Akr* mRNA levels profited from AMPK activity/intact phosphosites in Nrf2. Induction of all investigated target genes is predominantly dependent on Nrf2 activation in our model system, as evident by comparison of wt with isogenic Nrf2-deficient cells (Supplemental Fig. 7). The boosted induction of selected Nrf2 target genes in the presence of AMPK (i.e. *Hmox-1* versus *Gclc*) was not confined to Sfn, but recapitulated by xanthohumol, CDDO-Im or tert-butylhydroquinone, other well-known Nrf2 activators [28,29] (Supplemental Fig. 8).

Overall, AMPK-dependent phosphorylation seems to impart Nrf2 with increased transactivation potential for selected endogenous target genes, without altering nuclear translocation or general capability for inducing gene transcription.

## 4 Discussion

The main novel findings of this study are the association of AMPK activity with (i) Nrf2 phosphorylation at serines 374, 408 and 433 in living cells and (ii) enhanced expression of selected Nrf2 target genes.

We used label free MS-based analysis of immunoprecipitated Nrf2 from cells with activated and non-active AMPK to identify AMPK-dependent phosphosites. The highly variant degree of phosphorylation between independent biological replicates seen in the MS-based analysis, not unusual for phosphopeptides from replicates after a multi-step workup of independently processed samples (i.e. transfection, lysis, IP, digest, LC - MS runs on different days) of (comparably low abundant) proteins from live cells, hampers statistical significance. Nonetheless, the identified sites were consistently and reproducibly upregulated throughout the replicates upon growing AMPK activation (in experiments with both transfected human HEK cells and murine fibroblasts, as well as in the *in vitro* kinase assay). In contrast, S 215, a presumably AMPK-independent phosphosite in Nrf2, showed a basically unaltered phosphorylation degree irrespective of AMPK presence/activation. Therefore, we assume serines 374, 408 and 433 in Nrf2 to be phosphorylated in an AMPK-dependent manner in living cells. However, as our proteome data reached maximally 74% sequence coverage of Nrf2 (see Fig. 1) we cannot exclude the existence of other AMPK-dependent Nrf2 phosphosites in cells. For instance, the area around S 558, reported as *in vitro* AMPK phosphorylation site in Nrf2 [20], was not covered in our MS runs for immunoprecipitated Nrf2 (but confirmed in our *in vitro* assays, not shown). The use of targeted proteomic approaches like SILAC (stable isotopic label by amino acids in cell culture) could possibly increase the precision of obtained areas for phosphopeptides [30], but would demand more time and expenses as well as carry the risk of reduced proteome coverage impeding the use of the data sets for future interactome analyses [31]. To maximize sequence coverage, we already combined the generated data after digestion with LysC + trypsin or chymotrypsin (which theoretically should lead to a Nrf2 sequence coverage of >95% and cannot be markedly enhanced by the use of additional proteases as shown *in silico*, using ExPASy [32]). To augment number and quantity of detected phosphosites, enrichment via immobilized metal (e.g. Ti (IV+), Fe (III+) or Ga (III+)) affinity chromatography (IMAC) appears plausible. However, such strategies show selectivity towards single- or multi-phosphorylated peptides and might therefore manipulate the original relative phosphosite abundances, while still carrying the risk of contamination from non-phosphorylated peptides (e.g. acidic, charged, peptides) especially in more complex samples, such as whole cell lysates [33,34]. Thus we opted for a label free strategy, bypassing the need for metabolic or chemical labeling but relying on reproducible chromatographic separation to quantify peptides combined with an algorithm to match the results between several runs, and for enrichment on protein level of Nrf2 (GFP-Trap®) rather than on phospho peptides.

None of the here detected AMPK-dependent Nrf2 phosphosites complies with the consensus AMPK recognition motif [35,36]. That the phosphate transfer might still be directly catalyzed by AMPK was indicated by *in vitro* kinase assays in which gradually increasing AMPK activity went along with rising phosphorylation at serines 374, 408 and 433 in Nrf2. In contrast, S 215 phosphorylation did not respond to elevated AMPK activity at all. Moreover, mutual co-immunoprecipitation of AMPK and Nrf2 strongly supported the notion of their interaction in living cells, although it is no ultimate proof for direct interaction or phosphate transfer in the cell. Overall, Nrf2 could belong to the AMPK substrates not fulfilling the commonly accepted consensus motif [37]. Serine 374, 408 and 433 had already been known as phosphorylation sites of Nrf2 from other studies [PhosphoSitePlus database [38]], however, with no reference to AMPK so far. S 408 was reported to be phosphorylated by mitogen activated protein kinases (MAPK) (together with S 215, S 559 and S 577) [39], suggesting that this site may even integrate cues from different (stress) signaling pathways in the cell.

Concerning the biological relevance of the identified phosphosites, we could delineate, by comparison of EGFP-WT-with EGFP-TM-Nrf2, that they are not crucial for Nrf2 stability or general nuclear translocation and transactivating capacity in wt cells. Both EGFP-WT- and EGFP-TM-Nrf2 decayed comparably fast in HEK or wt MEF. The observed turnover for transfected Nrf2 in HEK cells occurred slower than reported for endogenous Nrf2 with a half-life of 20–30 min [40]. This might be explained by residual incompletely washed-out proteasome inhibitor MG132 or an overwhelmed endogenous degradation system in case of massively overexpressed EGFP-fusion protein of Nrf2 in these cells. Both EGFP-WT- and EGFP-TM-Nrf2 showed a longer half-life upon addition of an AMPK activator, suggesting that AMPK signaling prevents Nrf2 degradation, irrespective of the presence of the phosphosites. Plausible steps in the AMPK-dependent stabilization could be increased p62-mediated autophagy of Keap1 or impeded GSK3-β/β-TrCP-launched degradation. Interestingly, only in front of a Keap1-negative background EGFP-TM-Nrf2 showed a longer half-life than EGFP-WT-Nrf2 and is not further stabilized by activated AMPK. These findings prompt the intriguing hypothesis that the identified phosphosites (or one of them) prime Nrf2 for phosphorylation by GSK3β and subsequent β-TrCP-launched degradation. In this model, phosphorylation at S 374, 408 or 433 renders EGFP-WT-Nrf2 a proper substrate for GSK3β. AMPK activation, however, leads to inhibition of GSK3 enzymatic activity, which interferes with the β-TrCP-degradation pathway and finally causes Nrf2 stabilization. The Nrf2 mutant form lacks the priming phosphorylation sites, is thus less susceptible to GSK/β−TrCP-triggered decay and resistant to AMPK-mediated stabilization via GSK3 inhibition. This model deserves further detailed investigation and unambiguous corroboration in the future. In Keap1-positive cells, mainly used in this study, Nrf2 abundance is predominantly determined by Keap1 with minimal impact of β-TrCP and the triple mutation.

Mutation of the serine to alanine residues did not either affect nuclear accumulation of Nrf2 or induction of an ARE-dependent reporter gene after Sfn exposure. However, the triple mutant differentially influenced induction of selected endogenous Nrf2 target genes. Whereas *Hmox1* and *Akr1c14* displayed reduced inducibility by EGFP-TM-Nrf2, *Gclc* and *Nqo*1 showed comparable responses to Sfn-activated EGFP-WT- and EGFP-TM-Nrf2. This distinct inducibility of *Hmox1* and *Akr1c1*4 by EGFP-WT- and EGFP-TM-Nrf2 was copied by endogenous Nrf2 in AMPK wt and AMPKα1 −/− MEF, respectively, after activation of endogenous Nrf2 signaling by various stimuli. Four selected genes can only give a limited picture, and it cannot be completely ruled out at this point that (i) the transfected triple mutant or EGFP-fusion protein of Nrf2 displays any conformational or stoichiometric constraints for interaction with regulator proteins, or (ii) that AMPK-mediated signals other than Nrf2 phosphorylation influence Nrf2-dependent gene expression in the wt vs AMPKα1 −/− lineup. Those open questions warrant future clarification. Nonetheless, our data already strongly indicate that AMPK- mediated phosphorylation of Nrf2 enhances transactivation of a subset of Nrf2 target genes. This is already supported by a recent microarray analysis in wt and AMPK −/− cells showing that approximately only one third of the Sfn-induced Nrf2 target genes is susceptible to regulation by AMPK activation (own unpublished data). Notably, also the MAPK-dependent phosphorylations of Nrf2 seemed to exert a (mild) gene-selective effect [39], suggesting that Nrf2 phosphorylation, next to determining stability or nuclear residency [41], may be a mean to tweak the expression of certain Nrf2 targets according to which upstream kinase is activated. Such a gene selective influence of transcription factor phosphorylation has already been reported for the cyclin dependent kinase 5 (CDK5)-mediated modification of peroxisome proliferator activated receptor (PPAR)γ [42]. Future studies employing whole transcriptome analyses such as RNA Seq, GRO-Seq, or ChIP Seq, optimally in cell types of more relevance for studying detoxification and metabolism, such as hepatocytes, are warranted to get an unbiased holistic picture on which functional gene clusters within the Nrf2 transcriptome underlie the influence of AMPK and Nrf2 phosphorylation. Moreover, the signal relay from Nrf2 phosphorylation to finally boosted transactivation of only a subset of genes deserves further attention. It is conceivable that phosphorylation of Nrf2 alters binding to ARE sites, interaction with transcriptional coactivators/-repressors of Nrf2, including Maf, cAMP-binding protein (CBP), peroxisome proliferator-activated receptor-gamma coactivator (PGC) 1α, bromodomain protein Brd4 or Brahma related gene 1 (Brg1) [[43], [44], [45]], or recruitment of RNA polymerase. An entailed modification of the promoter complex composition and/or changed transactivation capacity may also distinctly affect different genes in a context-dependent manner, due to e.g. specific local chromatin organization, promoter organization and strength, number of ARE sites or dependence on additional enhancer elements. Future holistic studies digging into molecular details of the cooperativity between AMPK and Nrf2 are eagerly awaited. The interplay obviously goes beyond mere indirect stabilization of Nrf2 and may extend towards an exclusive fine-tuned expression of specific target genes by direct phosphorylation of Nrf2 by AMPK.

## Declaration of competing interest

None.
