# Supplementary material for: AMPK leads to phosphorylation of the transcription factor Nrf2, tuning transactivation of selected target genes
Source: Redox Biol. 2019 Nov 27;29:101393. doi: 10.1016/j.redox.2019.101393 (PMC6909106; doi:10.1016/j.redox.2019.101393)
Supplement: Multimedia component 2 [file mmc2.pdf]

SUPPLEMENTAL MATERIAL

AMPK leads to phosphorylation of the transcription factor Nrf2, tuning transactivation of selected target genes

Manuel Matzinger[1,2], Katrin Fischhuber[1], Daniel Pölöske[1], Karl Mechtler[2,3], Elke H. Heiss[1,*]

[1]Department of Pharmacognosy, University of Vienna, Vienna, Austria

[2]Institute of Molecular Pathology (IMP), Vienna BioCenter (VBC), Vienna, Austria

[3]Institute of Molecular Biotechnology, Austrian Academy of Sciences, Vienna BioCenter (VBC), Vienna, Austria

Supplemental Material

SBI-0206965, tert-BHQ and xanthohumol were obtained from Sigma. CDDO-IM was purchased from Tocris. A mammalian ubiquitin expression plasmid (#31815) was provided by Addgene, and the anti-Nrf2 (#1272), anti-ubiquitin (#3933) and anti- tubulin α/β (#2148) antibodies came from Cell Signaling.

Supplemental Figures 1-8 plus legends

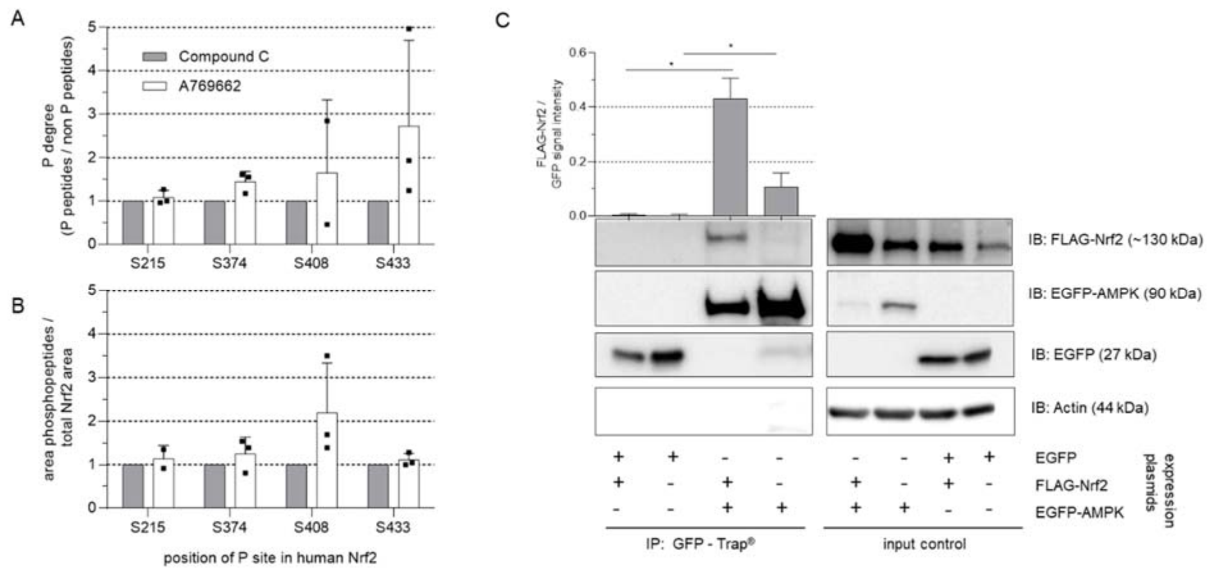

Supplemental Figure 1: **Nrf2 is AMPK-dependently phosphorylated at serine 374, 408 and 433 in HEK cells and co-precipitates with AMPK**. HEK cells were transfected with an expression plasmid for EGFP-tagged Nrf2 (EGFP-WT-Nrf2) and treated with MG132 and either with A769662 (50 µM, 4 h) or Compound C (10 µM, 4 h). Using GFP-Trap®, Nrf2 was pulled down and digested using chymotrypsin, analyzed via LC – MS and quantified using LFQ. The sum of the obtained areas for peptides with the indicated phosphosite was normalized to the sum of areas for the same peptides which are not phosphorylated (**A**) or to the total Nrf2 area in each sample (**B**). For S 408, after A769662 treatment, only phosphorylated peptides were quantified in one experiment, indicating an upregulation of this phosphosite. However, only 2 datapoints are shown in graph A, since division by zero is not possible. The obtained areas were normalized to Compound C treatment, 3 independent biological replicates were performed. **(C)** HEK cells were transfected with expression plasmids for Flag-Nrf2, EGFP-PRKAA1 (=EGFP-AMPK) or EGFP as indicated. After AMPK activation, stabilization of Nrf2 (via proteasome inhibition), lysis and pulldown via GFP-Trap®, eluates as well as an aliquot (20 µg protein) of the unprecipitated lysate (input control) were immunoblotted for EGFP-AMPK or EGFP (via α-GFP antibody) or (endogenous/transfected) Nrf2. Representative blots and compiled densitometric analyses (Nrf2 signal / GFP signal) are depicted (n = 3, mean + SD, unpaired Student's t-test, two tailed, $\alpha = 0.05$, * $P < 0.05$)

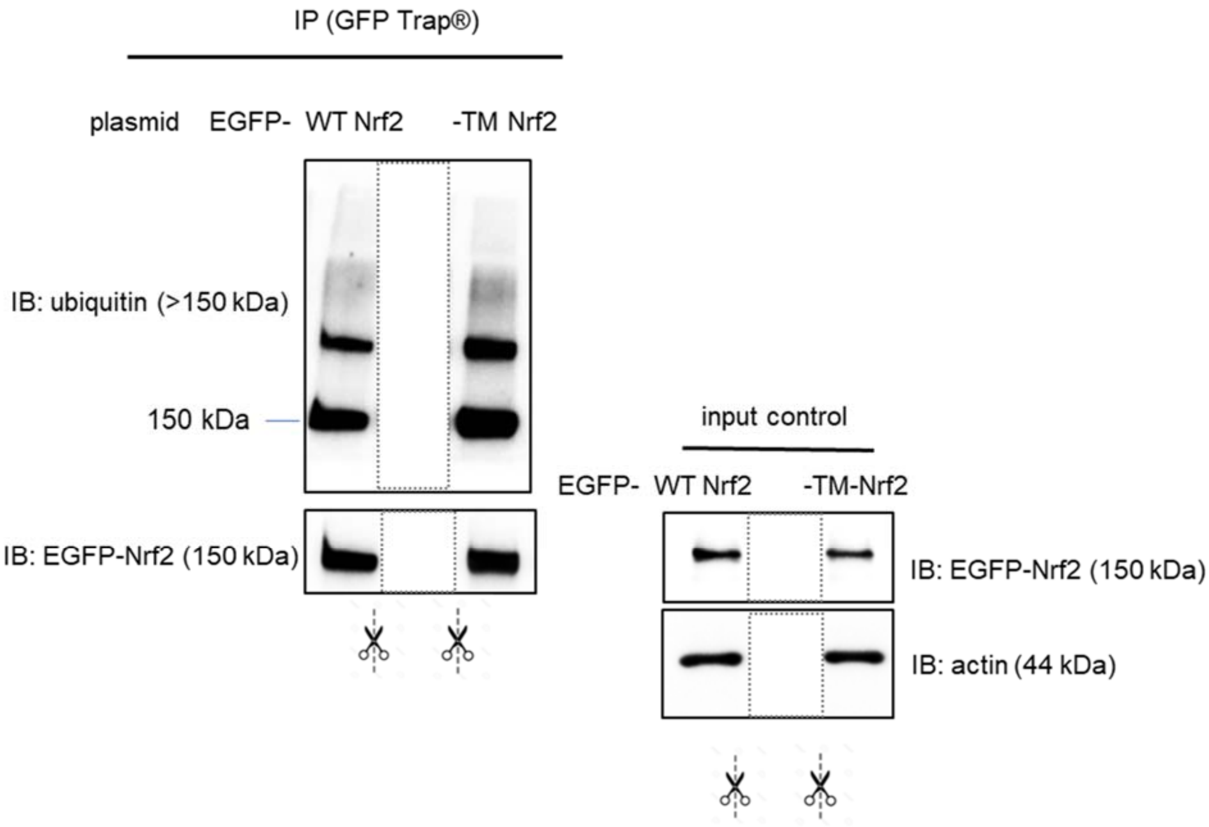

Supplemental Figure 2: WT and TM-Nrf2 show a comparable extent of ubiquitination.

MEF were transfected with EGFP-WT- or EGFP-TM-Nrf2 and an ubiquitin expression plasmid. After stabilization of Nrf2 (via proteasome inhibition), lysis and pulldown via GFP-Trap®, eluates and aliquots of unprecipitated lysates were immunoblotted for ubiquitin, WT- /TM-EGFP-Nrf2 (via α-GFP antibody) and actin. A representative blot out of three biological replicates is shown. Dashed lines + scissors indicate that the depicted lanes originate from the same membrane with interjacent bands (of no interest here) that were cut out/masked.

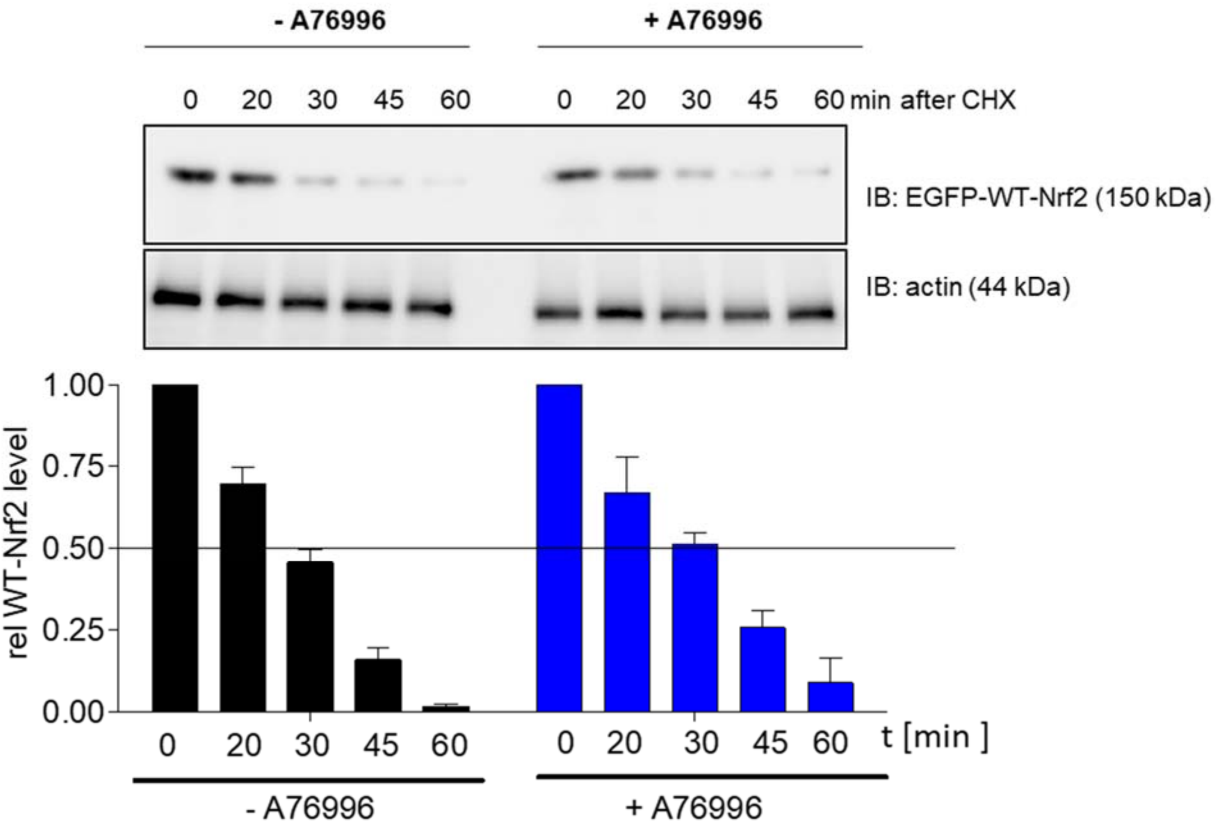

Supplemental Figure 3: A769662-mediated stabilization of Nrf2 blunted in AMPK -/- MEF. AMPKα1 -/- MEF were transfected with EGFP-WT-Nrf2 expression plasmid and treated with MG132 (10 µM) and DMSO or A769662 (50 µM) for 1 h. After washout of MG132 the translation inhibitor cycloheximide (30 µM) was added (time point 0) in the presence of A769662 (50 µM) (+) or DMSO (-) for the indicated periods of time. Cell lysates were subjected to immunoblot analysis for EGFP-WT-Nrf2 (via α-GFP antibody) or actin. Representative blots and compiled densitometric evaluations are depicted (n =3, mean + SD)

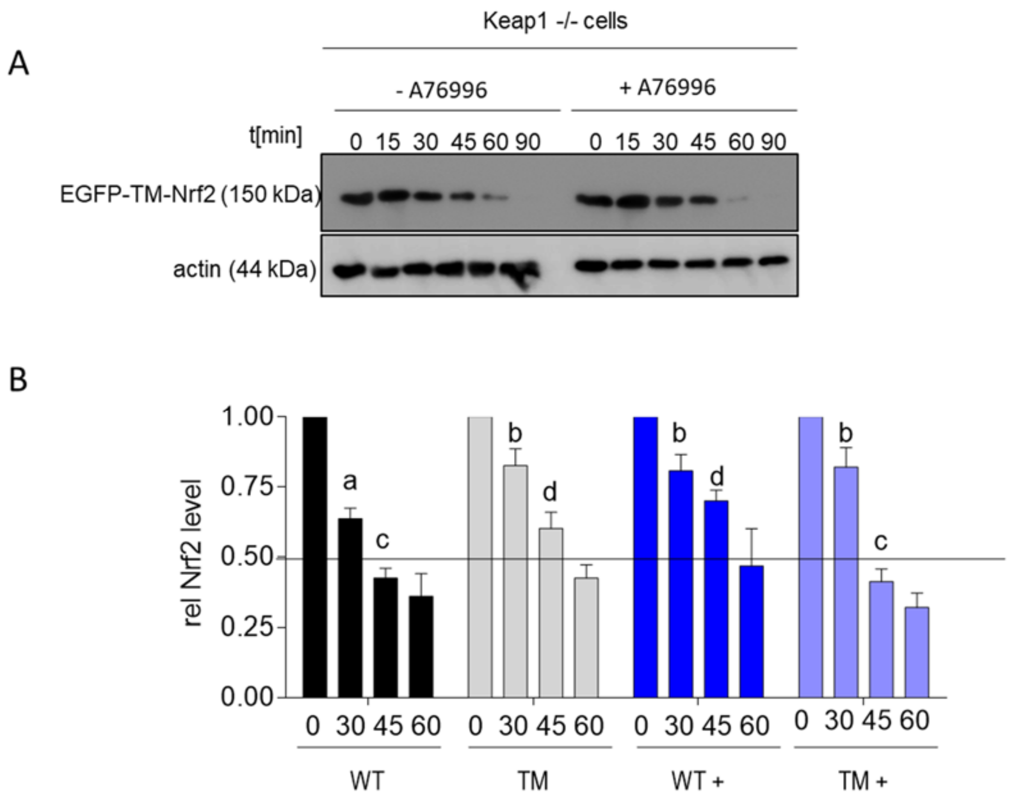

Supplemental Figure 4: In a Keap1-negative background, the TM mutant is not stabilized by AMPK activation and shows prolonged half-life compared to WT-Nrf2. (**A**) Keap1 -/- MEF were transfected with EGFP-tagged TM-Nrf2 expression plasmids, pretreated with A76996 (50 µM, 1h) as indicated and then exposed to cycloheximide (30 µM) for different periods of time. Cell lysates were subjected to immunoblot analysis for EGFP-TM-Nrf2 (via α-GFP antibody) or actin. Representative blots are shown. (**B**) Compiled densitometric evaluations of three independent experiments, also including the data for EGFP-WT-Nrf2 from Figure 4 of the main manuscript. EGFP-WT/TM-Nrf2 : actin ratios, related to signal at t = 0 are depicted (n = 3, mean + SD, unpaired Student's t-test, two tailed, α = 0.05, different superscript letters indicate differences with $p<0.05$).

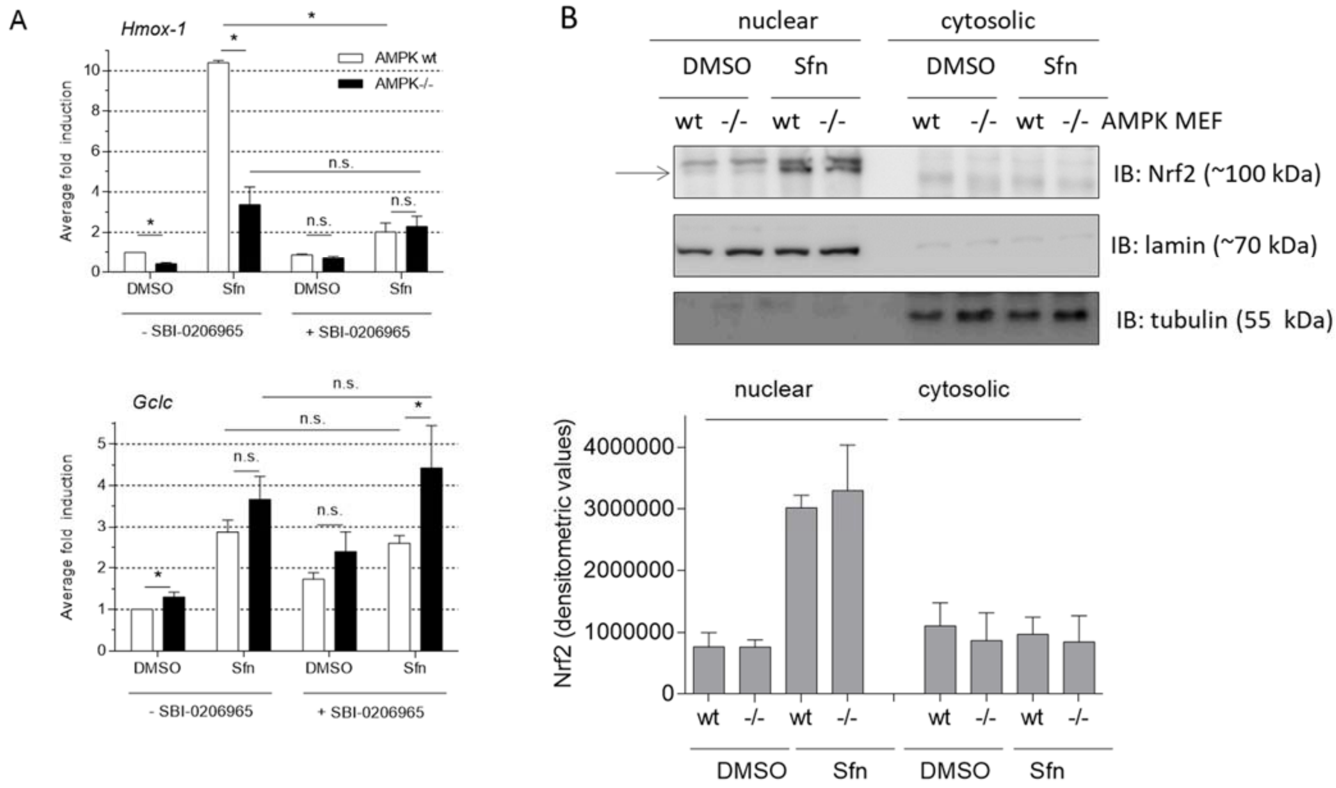

Supplemental Figure 5: The AMPK-dependent boost of *Hmox1* but not *Gclc* gene expression is confirmed by use of an AMPK inhibitor and not due to different Nrf2 nuclear abundance between WT and AMPK-/- MEF. (A) AMPK wt and -/- MEF were treated with Sfn (5 µM) or DMSO as vehicle control for 4 h in the presence or absence of the inhibitor SBI-0206965 (30 µM) as indicated. RNA was extracted and analyzed for abundance of *Hmox-1* or *Gclc* mRNA by qPCR (*Hprt* as reference gene). Bars depict compiled relative expression levels. (n = 3; mean + SD, unpaired Student's t-test, $\alpha = 0.05$, * $P \leq 0.05$, ns: not significant **(B)** AMPK wt and -/- MEF were treated with Sfn (5 µM, 2 h) prior to extraction of cytosolic and nuclear fraction and immunoblotting for endogenous Nrf2 (arrow), lamin and tubulin. Representative blot images and compiled data from three independent experiments are depicted.

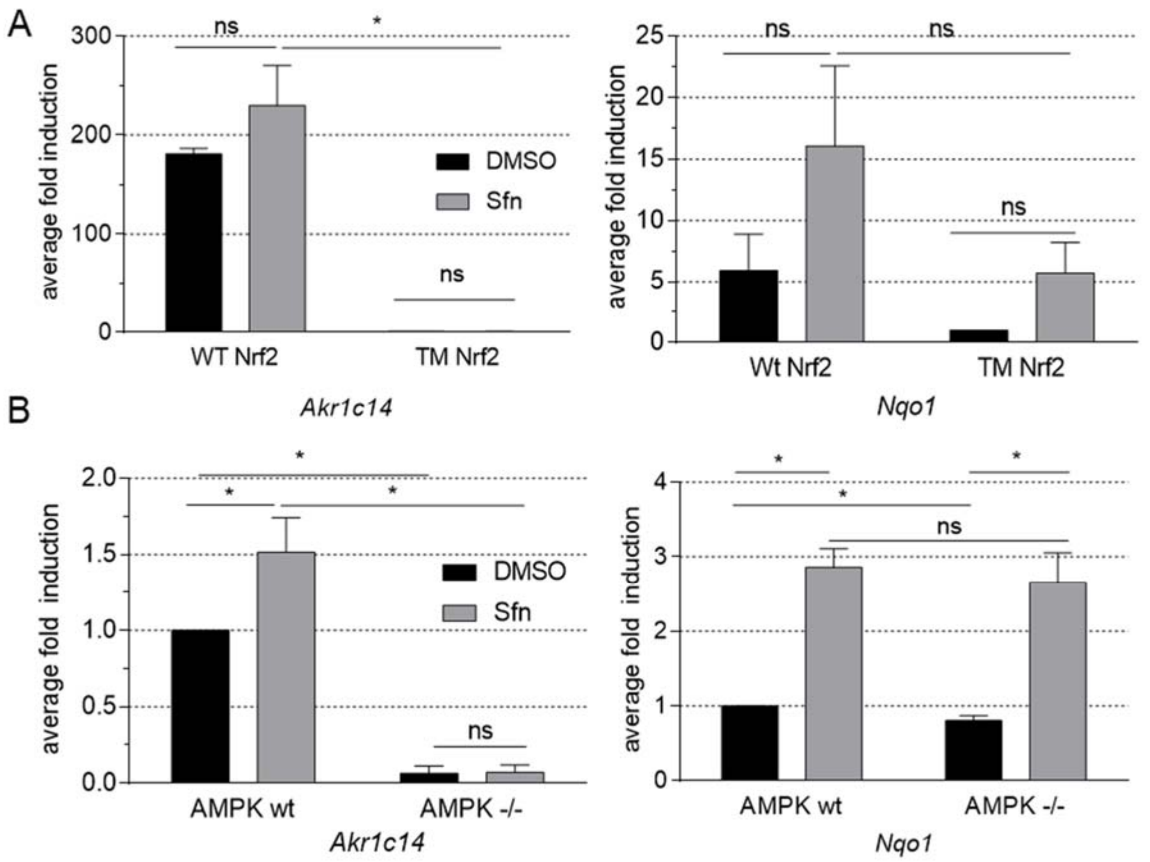

Supplemental Figure 6: AMPK-dependent phosphosites in Nrf2 enhance expression of selected endogenous Nrf2 target genes. MEF Nrf2 -/- cells transfected with EGFP-WT- or EGFP-TM-Nrf2 **(A)** and wt or AMPK$\alpha 1$ -/- MEF were treated with DMSO as vehicle control or sulforaphane (Sfn, 5 µM) for 4 h. **(B)** RNA was extracted and analyzed for abundance of *Akr1c14* and *Nqo1* mRNA by qPCR (*Hprt* as reference gene). Bars depict compiled relative expression levels. (n = 3; mean + SD, unpaired Student's or Welch's t-test, $\alpha = 0.05$, * $P \leq 0.05$, ns: not significant).

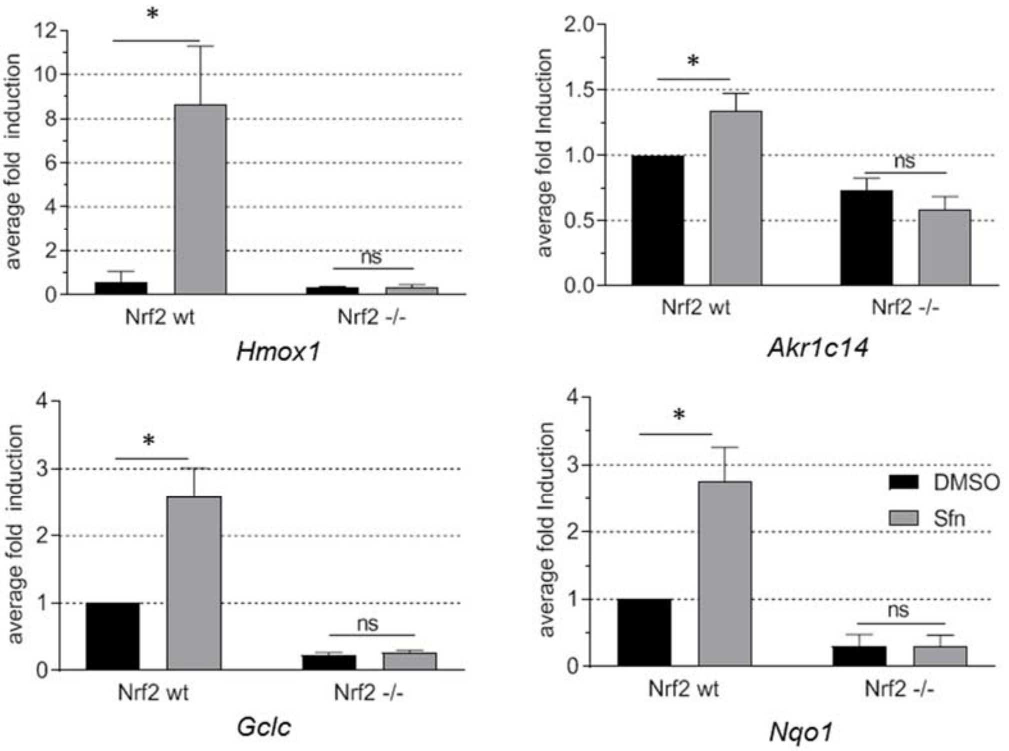

Supplemental Figure 7: Expression of selected endogenous Nrf2 target genes is Nrf2-dependent.

Nrf2 wt or -/- MEF were treated with DMSO as vehicle control or sulforaphane (Sfn, 5 µM) for 4 h. RNA was extracted and analyzed for abundance of *Hmox1*, *Akr1c14*, *Gclc* and *Nqo1* mRNA by qPCR (*Hprt* as reference gene). Bars depict compiled relative expression levels. (n = 3; mean + SD, unpaired Student's or Welch's t-test, α = 0.05, * P ≤ 0.05, ns: not significant).

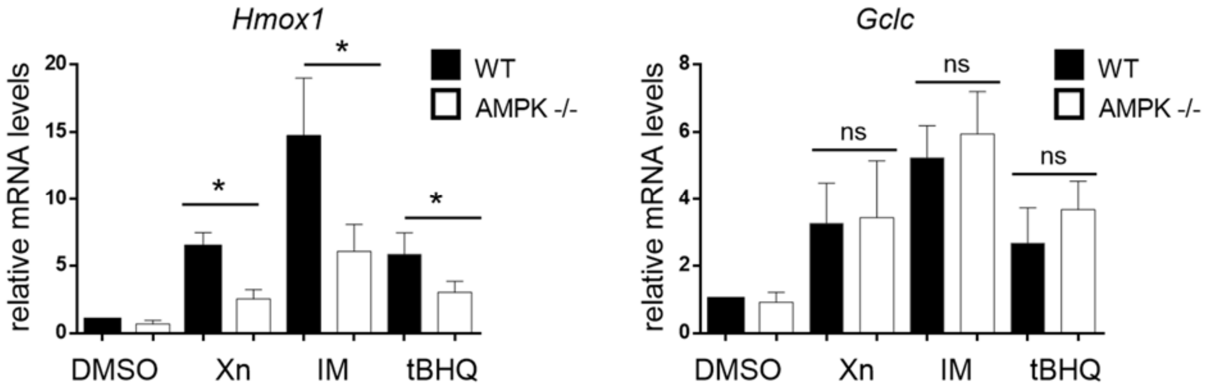

Supplemental Figure 8: Reduced induction of *Hmox* (but not *Gclc*) gene expression in AMPK-/- MEF is not confined to Sfn, but extended to other Nrf2 activators. AMPKα wt or -/- MEF were treated with DMSO as vehicle control (D), xanthohumol (Xn, 5 µM), CDDO-IM (IM 30 nM) or tert-butylhydrochinone (tBHQ, 15 µM) for 4 h. RNA was extracted and analyzed for abundance of *Hmox1*, and *Gclc* mRNA by qPCR (*Hprt* as reference gene). Bars depict compiled relative expression levels of three independent experiments. (* $P \leq 0.05$, ns: not significant)
